# Improve Solubility and Develop Personalized Itraconazole Dosages via Forming Amorphous Solid Dispersions with Hydrophilic Polymers Utilizing HME and 3D Printing Technologies

**DOI:** 10.3390/polym16233302

**Published:** 2024-11-26

**Authors:** Lianghao Huang, Jingjing Guo, Yusen Li, Weiwei Yang, Wen Ni, Yaru Jia, Mingchao Yu, Jiaxiang Zhang

**Affiliations:** 1 Key Laboratory of Marine Drugs, Ministry of Education, School of Medicine and Pharmacy, Ocean University of China, Qingdao 266003, China; huanglianghao1@outlook.com (L.H.); jingjingguo1208@outlook.com (J.G.); 19511639685@163.com (Y.L.); niwen0716@outlook.com (W.N.); jiayaru009@outlook.com (Y.J.); yumingchao11@outlook.com (M.Y.); 2 Pharmaceutical Products Research and Development Center, Marine Biomedical Research Institute of Qingdao, Qingdao 266137, China; 3 Qingdao Institute of Bioenergy and Bioprocess Technology, Chinese Academy of Sciences, Qingdao 266101, China; yangweiwei0125@gmail.com; 4 Shandong Energy Institute, Qingdao 266101, China; 5 Qingdao New Energy Shandong Laboratory, Qingdao 266101, China

**Keywords:** programmed drug release, amorphous solid dispersion, 3D printing, hot-melt extrusion, Itraconazole

## Abstract

Itraconazole (ITZ), a broad-spectrum triazole antifungal agent, exhibits remarkable pharmacodynamic and pharmacokinetic properties. However, the low solubility of ITZ significantly reduces its oral bioavailability. Furthermore, it has been reported that this medication can result in dose-related adverse effects. Therefore, the objective of this study was to enhance the solubility of ITZ through the utilization of various polymers and to manufacture personalized and programmable release ITZ tablets. Five different polymers were selected as water-soluble carriers. Thirty percent *w*/*w* ITZ was mixed with seventy percent *w*/*w* of the polymers, which were then extruded. A series of physical and chemical characterization studies were conducted, including DSC, PXRD, PLM, and in vitro drug release studies. The results demonstrated that ITZ was dispersed within the polymers, forming ASDs that markedly enhanced its solubility and dissolution rate. Consequently, soluplus^®^ was employed as the polymer for the extrusion of ITZ-loaded filaments, which were subsequently designed and printed. The in vitro drug release studies indicated that the release of ITZ could be regulated by modifying the 3D structure design. Overall, this study found that the combination of HME and 3D printing technologies could represent an optimal approach for the development of personalized and precise drug delivery dosages.

## 1. Introduction

Fungal infection, or mycosis, is a disease caused by fungi that affects superficially, subcutaneously, and systemically. It is a common disease distributed world-wide, by which more than 1 billion people are affected each year, and there were around 1.7 million deaths caused by fungal disease in 2020 [1,2,3,4]. Superficial and subcutaneous fungal infections usually only affect the skin, hair, and other external parts of the body, causing no serious health issues [5,6], while systemic fungal infections are more serious, such as histoplasmosis caused by histoplasma capsulatum, which spreads throughout the lungs and other organs and can be fatal if untreated [7]. Fungal infections can be treated with topical or systemic antifungal agents depending on the extent of the infection. For example, severe histoplasmosis cases may require treatment with systemic antifungal drugs, typically amphotericin B followed by oral itraconazole [8]. In acute pulmonary histoplasmosis, 6 to 12 weeks of treatment may be sufficient, but in severe cases, itraconazole (ITZ) treatment may need to be continued for at least a year [9].

ITZ is in the triazole family of antifungal medication used to treat fungal infections including histoplasmosis, and it inhibits the synthesis of ergosterol mediated by cell membrane pigment P450 oxidase [10]. Because fungal cells are eukaryotes like humans, substances that are toxic to fungi are usually harmful to humans as well. Antifungal agents usually work through lipids (ergosterol, which is different from animal cholesterol) on the fungal cell membrane. Usually, ITZ is a relatively well-tolerated drug, and its known side effects include nausea, vomiting, abdominal pain, fatigue, loss of appetite, jaundice, itching, dark urine, pale stool, and headache. However, there are reported serious side effects such as elevated alanine aminotransferase levels (4% of patients taking ITZ), liver failure, and sometimes fatal and congestive heart failure (1/10,000) [11,12]. Additionally, ITZ is a weak basic and has low solubility but high permeability; it is a class II drug under the biopharmaceutical classification system (BCS). Poor water solubility resulting in the oral bioavailability of ITZ is around 55% maximal if taken with a full meal [13]. In addition, patients with different weights, ages, genders, or races react differently when taking the same marketed ITZ dosages. So, there is an urgent need to develop a novel ITZ dosage with improved solubility and bioavailability as well as personalized drug delivery of ITZ.

Forming amorphous solid dispersions (ASDs) is one of the highly effective methods for enhancing the solubility and dissolution rate of poorly water-soluble medications, ultimately increasing their bioavailability [14,15]. The amorphous form of a drug is characterized by a disorganized structure possessing a large free-energy force. Because of this unique structural feature, the apparent water solubility, dissolution rate, and oral absorption are enhanced. Moreover, the amorphous phase holds an energetic advantage. Specifically, since there is no need for energy to break the crystalline structure during the process, it can more easily exhibit its superior properties related to solubility and absorption compared to the crystalline form of the drug. Generally, the active pharmaceutical ingredient (API) is encapsulated and maintained in a polymer matrix in an amorphous state. This indicates that the polymer matrix is crucial in enhancing the physical and solution thermodynamic stability of ASD systems [16,17]. Various techniques have been widely used by industries or researchers to produce ASDs including an array of solid state, mechanochemical, and liquid assisted techniques [18,19]. Nevertheless, only a few methods have demonstrated the potential for producing scalable, high-quality ASDs through the use of green chemistry techniques.

Nowadays, hot-melt extrusion (HME) is widely used for a broad spectrum of applications in the pharmaceutical field [20,21,22]. Several drug products made by HME have been approved by the US Food and Drug Administration (FDA) and used in clinics, suggesting the huge potential of HME in pharmaceutical manufacturing [23]. HME is a process that uses heat, pressure, and high sheer in a channel to transform raw materials into a uniform product. This method was proven to be effective in enhancing the thermodynamic solubility of APIs with poor water solubility [24,25]. In contrast to traditional ASD preparation methods, HME provides numerous benefits such as processing without organic solvents, quick volumetric heating, energy efficiency, and cost effectiveness [26]. The preparation of ASDs using HME is a key step in the push to integrate continuous manufacturing (CM) principles in the pharmaceutical industry as well [27,28,29].

Furthermore, precision medicine has become a hot research trend and gained a huge amount of interest from patients, researchers, and caregivers [30]. Three-dimensional printing, formally designated as additive manufacturing (AM), allows the on-demand manufacturing of personalized and programmable released dosages, which could be optimal for precision drug delivery. Three-dimensional printing is a process that assembles objects in a layer-by-layer fashion, utilizing computer-aided designs [31]. The computer-aided design of 3D models based on an individual’s age, height, weight, and other characteristics allows for the printing of dosages with varying structures, thus enabling the implementation of precision medicine [32]. Among the various AM technologies, fused depositional modeling (FDM), stereolithography, selective laser sintering (SLS), material jetting, and binder jetting are most frequently utilized in the pharmaceutical domain. Notably, FDM has emerged as the most prevalent AM technology in the pharmaceutical sector [33]. FDM-3D printing technologies typically employ a drug-loaded filament as the initial material, which is subsequently melted by the hot end and distributed on the building platform in successive layers. An increasing number of reports demonstrate the potential integration of HME and 3D printing technology as a continuous process, showcasing the respective advantages such as the facilitation of the manufacture of more intricate structural dosing forms and personalized drug products as well as the improvement of solubility and bioavailability for poorly water-soluble drugs [34,35]. However, even though more and more publications work on combining HME with FDM-3D printing, the mechanisms of improving solubility and bioavailability as well as achieving personalized drug delivery have not yet been explained well.

In HME and FDM-3D printing processes, materials with outstanding fluidity, thermal stability, and machinability are essential [36]. Consequently, the selection of polymer materials is of paramount importance. Carbohydrates, celebrated for their natural profusion and diversity, play a pivotal role in the domain of formulations [37,38,39]. The carbohydrate polymer materials used in HME and FDM-3D printing mainly include cellulose, starch, chitosan, and dextran, among others [40,41,42,43,44,45]. These can serve as the substrate or carrier for drugs. The structure and properties of carbohydrates exert an influence on the diffusion and release of drugs. For instance, highly branched-chain carbohydrates may offer slower release owing to their more complex structure, while linear carbohydrates may lead to faster release [46,47,48]. The porosity and crystallinity of carbohydrate-based materials can also be improved by HME and FDM-3D printing, further impacting drug release [49]. Moreover, carbohydrates can offer great biocompatibility and biodegradability, which render them highly suitable for applications in pharmaceutical fields [50,51]. Thus, this combination holds remarkable promise for the development of personalized drug delivery systems and relevant advanced applications.

So, in this work, pre-formulation and formulation studies of preparing ITZ ASDs with various polymers were carried out. Process development including both HME and 3D printing were conducted. And a series of physical and chemical characterizations of raw materials, extrudates, and 3D-printed dosages were also conducted. The differential scanning calorimetry (DSC), powder X-ray diffraction (PXRD), and hot-stage polarized light microscopy (PLM) results demonstrated that the extrusion process resulted in the transformation of the initial material into an ASD. The in vitro study demonstrated that the dissolution rate and degree of most extrudates were superior to that of the physical mixture (PM) group. Moreover, soluplus^®^ was demonstrated to markedly enhance the solubility and dissolution rate of ITZ in comparison to other polymers. Consequently, five ITZ tablets with distinct structural designs were successfully manufactured using 3D printing technology. In order to facilitate a comparison, direct compression tablets of the extrudates (EXT) were prepared. This study used novel approaches to improve the solubility of poorly water-soluble drugs and offers insights into precision drug delivery via programmable release.

## 2. Materials and Methods

### 2.1. Pre-Formulation and Solid-State Studies

#### 2.1.1. Materials

**Figure 1:**
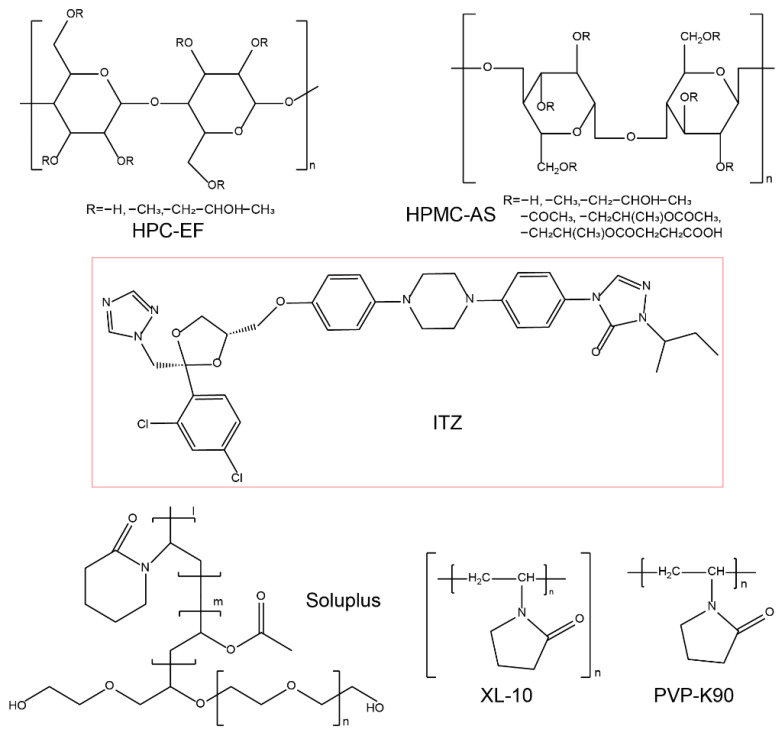
The 2D chemical structures of polymers used and the ITZ.

Itraconazole (ITZ) was purchased from Macklin Biochemical Co., Ltd. (Shanghai, China). Hydroxypropyl methylcellulose acetate succinate (HPMC-AS) and hydroxypropyl cellulose (HPC) are cellulose-based polysaccharides with a linear chain of β(1→4)-linked D-glucose units. HPMC-AS contains 4 randomly substituted groups on the hydroxyl group; the mass percentages are: methoxy group, 12-18%; hydroxypropyl group, 4–23%; acetyl group, 2–16%; and succinyl group, 4–28%. The HPMC-AS (10,000–500,000 Da) used in this work was donated from Taian Ruitai cellulose Co., Ltd. (Taian, China). HPC is an ether of cellulose in which the hydroxyl groups on the glucose units are replaced by hydroxypropyl groups. Soluplus^®^ is a polyvinyl caprolactam-polyvinyl acetate-polyethylene glycol graft copolymer, in which the polyvinyl caprolactam-polyvinyl acetate side chains are attached to a polyethylene glycol backbone that allows its amphiphilic properties [52]. Polyvinylpyrrolidone (PVP), or povidone, is a linear hydrophilic polymer containing N-vinylpyrrolidone as the monomer; the crosslinked polyvinylpyrrolidone, crosslinked PVP, is insoluble but with strong interfacial activity and can improve the solubility of poorly soluble drugs. Soluplus^®^ (molecular weight: 9000–14,000 Da) was purchased from BASF (Ludwigshafen, Germany). Klucel^TM^ EF PHARM (HPC-EF, 80,000 Da, (10% solution 300–600 mPas)), Plasdone K-90 (PVP-K90, K valve: 85–95, 1,300,000 Da, 55.0 mPas), and Polyplasdone XL-10 (XL-10, particle size: 25–40 μm) were donated from Ashland^®^. The chemical structures are shown in Figure 1. Diluted water was used for all solutions and formulations. All other chemicals, solvents, and reagents used in this work were either analytical or HPLC grades.

#### 2.1.2. Solid States’ Analysis

##### Thermalgravimetric Analysis

A TGA (Netzsch TG 209 F3, Selb, Germany) was used to obtain the thermal degradation information of ITZ, polymers, and PM. Samples of 5–10 mg were loaded onto the sample pan and then heated from 20 °C to 400 °C under the ultra-purified nitrogen-purged condition. Microsoft Excel was used to collect and analyze the data (version 2310 Build 16.0.16924.20054).

##### Differential Scanning Calorimetry

DSC 200 F3 equipment (NETZSCH Geratebau GmbH, Selb, Germany) was used to obtain the melting properties of ITZ, polymers, PM, and EXT. Samples of 5–15 mg were weighed and transferred to standard aluminum pans, which were sealed using standard aluminum lids (DSC consumables incorporated, Austin, TX, USA). The analysis was performed at a temperature range of 20 °C to 200 °C, with a ramp rate of 20 °C per minute.

##### Powder X-Ray Diffraction

The crystallinity of ITZ, PM, and EXT was investigated using a benchtop PXRD instrument (D/max-2200PC, Rigaku Corporation, Tokyo, Japan). Briefly, the samples were loaded onto the sample cells, which were then placed in the sample holder where the samples were scanned from a 2θ angle of 5° to 5° with a scan speed of 2°/min, scan step of 0.02°, and a scan resolution of 0.0025. The current and voltage of the system were maintained at 15 mV and 45 V, respectively. The collected data were plotted as an overlay graph of 2θ versus intensity.

##### Hot-Stage Polarized Light Microscopy

A CX40P polarized photomicroscope (Ningbo ShunYu Analytical Instrument Co., Ltd., Yuyao, China) equipped with a hot stage (Linkam Scientific Instruments Ltd., Salfords, UK) was used to investigate the melting behaviors and the residual crystalline ITZ in the extruded samples. The drug and the milled samples were spread out evenly onto a glass slide and any excess powder was dusted off. A coverslip was placed on the sample slides and the slide was then placed onto the microscope stage and observed under a 10X magnification. Birefringence, a property observed in crystalline substances, was observed in all the samples. Images were captured using a special digital camera (EP-SUF880, Markham, ON, Canada) under light and dark background conditions using a 530 nm compensator (U-TP530, Olympus Corporation, Shinjuku City, Tokyo, Japan).

#### 2.1.3. Qualification and Quantification of ITZ Using HPLC

The amount of released ITZ was determined by HPLC (Agilent 1260 Infinity II, Santa Clara, CA, USA) and analyzed using Agilent DAD software (Version C.01.07 SR2 [255], Agilent Technologies, Inc., Santa Clara, CA, USA). Samples (10 μL) were injected into a C18 column (Diamonsil ^®^ Plus 5 µm C18, 250 × 4.6 mm, DiKMA^®^, Beijing, China) and eluted with water/acetonitrile (80:20 *v*/*v*) as the mobile phase. The flow rate was 1.0 mL/min and the detection wavelength was at 262 nm. The data are presented as average ± standard deviation of three experiments and analyzed using Excel (version 2019).

#### 2.1.4. Equilibrium Solubility Measurement

Saturated solubility values of ITZ simulated gastric fluid (USP SGF, lacking pepsin) (hydrochloric acid, pH 1.2) were determined at 37 ± 0.5 °C. The experiments involved adding excess ITZ (0.1 g) to 20 mL of solvent. The shaker was set to 300 rpm with an equilibrium time of 24 h. After the experiment, each vial was taken out of the shaker and the undissolved particles were allowed to settle for 24 h. Once all solid particles settled, the supernatants were carefully removed, diluted, and analyzed using the HPLC method to quantify ITZ. Each experiment was repeated three times (n = 3.0) for accuracy. Statistical significance was computed via GraphPad Prism 9.4 (San Diego, CA, USA) through one-way ANOVA analysis, followed by post hoc Tukey’s tests.

### 2.2. Formulation Studies

#### 2.2.1. Preparation of the ASDs

**Figure 2:**
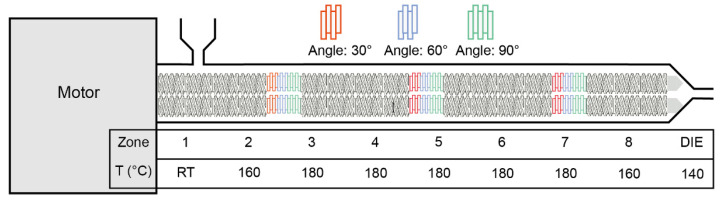
The demonstration of screw design and extrusion set-ups.

In this study, various formulations were investigated. For all formulations, 30% *w*/*w* of ITZ was mixed with 70% *w*/*w* of each polymer and marked as physical mixture, PM. Prior to formulation, both ITZ and polymers underwent desiccation in a vacuum oven at 50 °C for 24 h to remove moisture. An 11 mm corotating twin screw extruder (Thermal Fisher Scientific Process 11 HYG, Waltham, MA, USA) with eight individual heating zones and a 2 mm round die was used for the preparation of the ITZ-loaded filaments. The PM was manually fed into the feeding zone, and the feeding rate was controlled at around 3 g/min while the extrusion speed was set at 50 rpm. The screw configuration and extrusion temperature set-ups are demonstrated in Figure 2. Throughout the extrusion process, melting temperature, torque, and die pressure were closely monitored and recorded. Filaments were collected and then ground, while the ground particulates were sieved through the 40-mesh screen and labeled as extrudate, EXT. Both the PM and EXT samples were stored in a validated desiccator appropriately for subsequent assessment.

#### 2.2.2. Characterization of EXT

##### Solid States’ Analysis

DSC, PLM, and PXRD studies were carried out to investigate if the ITZ in EXTs stayed in crystalline form or transformed into amorphous states. Detailed experimental methods were mentioned in the Sections from “Thermalgravimetric Analysis” to “Hot-Stage Polarized Light Microscopy.

##### Solubility of EXTs’ ITZ

Saturated solubility values of PM and EXT in simulated gastric fluid (USP SGF, lacking pepsin) (hydrochloric acid, pH 1.2) were determined at 37 ± 0.5 °C as well. The experiments were conducted as described in Section 2.1.4.

##### In Vitro Drug Release from the EXTs

Drug release from the powders was determined using a Chinese pharmacopeia (ChP)-II dissolution apparatus (RC8MD, TIANDA TIANFA, Tianjin, China). The dissolution tests were conducted according to the United States Pharmacopeia standards using simulated gastric juices (USP SGF, without pepsin) (hydrochloric acid, pH 1.2), which represent human simulated gastric juices. Precisely weighed amounts of ITZ (9 and 100 mg), PM (equivalent to 9 and 100 mg ITZ), and EXT (equivalent to 9 and 100 mg ITZ) were added to in triplicate using 900 mL of the dissolution medium at 37 ± 0.5 °C for 6 h. The paddle speed was set at 100 rpm. Samples were taken at 5, 15, 30, 45, 60, 90, and 120 min for HPLC analysis (methods described in Section “Qualification and quantification of ITZ using HPLC)”.

### 2.3. 3D Designs and Printing

**Figure 3:**
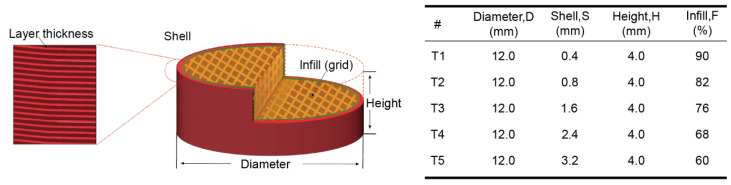
The demonstration of the 3D design and the design parameters of the tablets.

All tablets were designed with a cylindrical-shaped 3D builder software (Version 18.0.1931.0, Microsoft Corporation, Redmond, WA, USA). As shown in Figure 3, the designed models were sliced into different tablet designs with different shell thicknesses, layer heights, and infill densities using CURA software (Version 5.2.1, Ultimaker, Utrecht, The Netherlands). All the tablets were 100% opened at the bottom and top. An Ender-3 S1 Pro printer (Shenzhen Creality 3d Technology Co., Ltd., Shenzhen, China) with a 0.4 mm printing nozzle was used to produce the designed tablets. All the tablets were printed under the same printing conditions, and the printing temperature and building bed temperature were set at 180 °C and 50 °C, respectively. The printing speed was set at 20 mm/s. The EXT groups were compressed in a tablet press machine (Nuzhen Technology Co., Ltd., Shanghai, China) at 100 bars, resulting in 400 mg tablets each. The diameter and thickness of the tablets were determined using a VWR^®^ digital caliper (VWRI819-0013, Radnor, PA, USA). The printed tablets were imaged using a dino-Lite optical microscope.

### 2.4. In Vitro Drug Release from the Printed Tablets

The drug dissolution rates of tablets with each designed 3D structure were evaluated. Each printed tablet contained 120 mg of ITZ. The drug release was also conducted as USP SGF without pepsin (hydrochloric acid, pH 1.2), using a Chinese pharmacopeia (ChP)-II dissolution apparatus. Detailed experimental methods are mentioned in the Section “In Vitro Drug Release from the EXTs”.

### 2.5. Release Kinetic Studies of Printed Tablets

Zero-order model, first-order model, Higuchi model, Korsmeyer–Peppas model, and Peppes–Sahlin model were used to fit the dissolution kinetics [53]. Correlation coefficient (R^2^) was used to assess the accuracy of each model.

## 3. Results and Discussions

### 3.1. Solid States’ Analysis Studies

#### 3.1.1. TGA Studies

**Figure 4:**
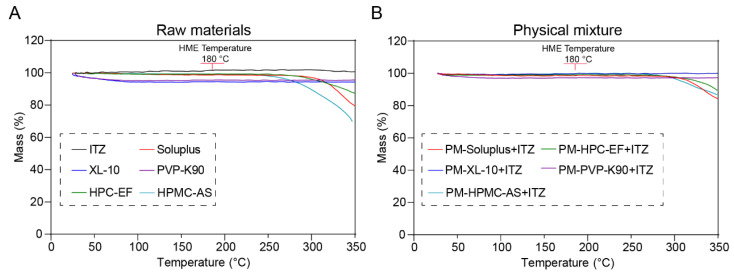
The thermogravimetric curves of raw ITZ, HPMC-AS, HPC-EF, PVP-K90, XL-10, Soluplus^®^ (**A**) and the respective PM (**B**).

The thermal stability of materials must be ensured during the thermal process to avoid potential thermal degradation, especially in this study where hot-melt extrusion was carried out at relatively high temperatures. Both raw materials and PM were heated to 350 °C at a rate of 20 °C/min. As shown in Figure 4A, ITZ showed no degradation trend within the temperature range of 350 °C. This indicates that, within this temperature interval, the ITZ maintained a relatively stable performance, while the polymers showed different initial thermal degradation temperatures, varying from 230 to 300 °C. The PM showed a relatively higher initial degradation temperature (270 to 320 °C) compared to the ITZ, which might be because the ITZ dissolved into the polymer matrix during the ramp. So, in the following investigation, both the HME and 3D printing process were performed at a maximum temperature of 180 °C in order to avoid potential thermal degradations.

#### 3.1.2. DSC Analysis

**Figure 5:**
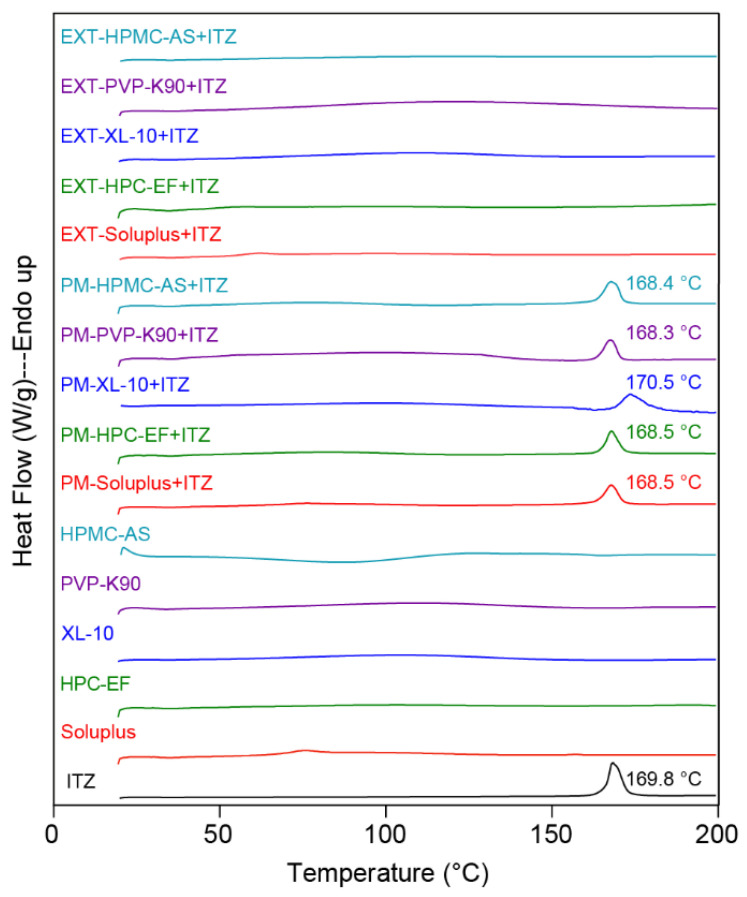
The DSC curves of raw materials, physical mixtures, and HME extrudates.

The DSC experiments were carried out to analyze the crystalline nature of raw ITZ, as well as HPMC-AS, HPC-EF, PVP-K90, XL-10, and soluplus^®^, along with their respective PM and EXT. As illustrated in Figure 5, a sharp endothermic peak was observed at 169.8 °C for ITZ, which aligns closely with the reported melting point [54]. Moreover, the PM exhibited smaller endothermic peaks compared to the raw ITZ and the PM peaks were also slightly shifted to the lower temperature. This may have been due to part of the crystalline ITZ dissolving into the polymeric matrix before reaching the melting temperature of the ITZ and the remaining crystalline ITZ melting around its intrinsic melting point, resulting in the attenuated endothermic peak in PMs. Additionally, such attenuated peaks also indicate that the IR might have formed ASD during the extrusion process. In order to make all crystalline ITZ transfer to amorphous states and form ASD with selected polymers, the HME process temperature was set at around 10 °C above the melting point of ITZ.

The absence of a distinct endothermic peak in the EXT during the heating process indicates the potential dissolution or dispersion within each polymer prior to reaching its melting point. Additionally, the absence of a distinct endothermic peak in the EXT also indicates that ITZ was miscible with the polymers and could form ASDs during the extrusion process. However, the transformation of ITZ from a crystalline to an amorphous state during the thermal treatment could not be definitively confirmed due to limitations in instrument sensitivity and detection thresholds. Instruments of relatively low sensitivity cannot detect subtle structural alterations. Additionally, if the magnitude of the transformation is under the detection threshold, it will not be detected. Further analyses using PXRD and PLM will be detailed in subsequent sections to verify the crystallinity of each extruded sample.

#### 3.1.3. Hot-Staged PLM

**Figure 6:**
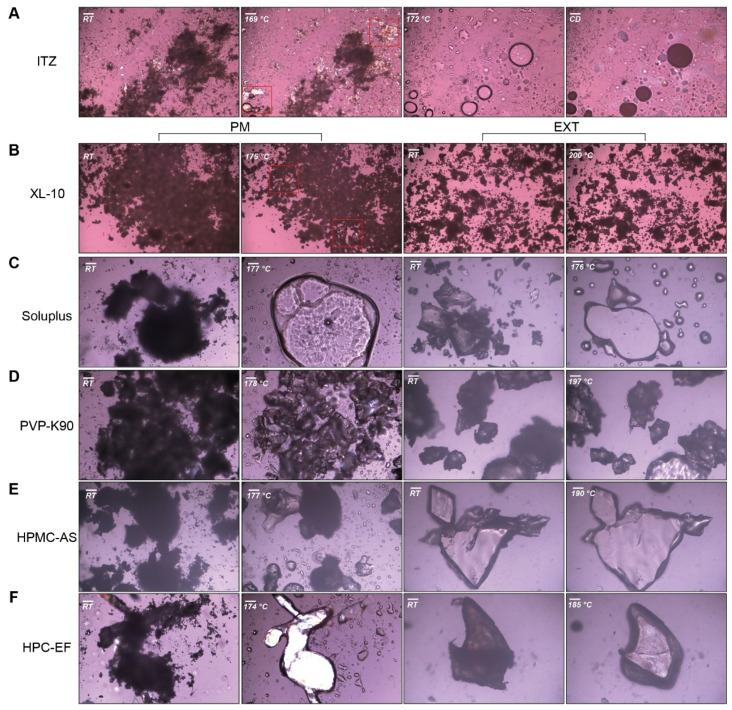
Hot-stage PLM pictures of heat each formulation until melt: (**A**) ITZ; (**B**–**F**) PM and EXT group of XL-10, soluplus^®^, PVP-K90, HPMC-AS, and HPC-EF. Scale bar: 100 μm.

A hot-staged PLM analysis was conducted to obtain crystalline form transformation during the heating process. Images of polymers, ITZ, and PM observed through PLM are displayed in Figure 6. As demonstrated in Figure 6A, the optically birefringent characteristics of ITZ were examined under polarized light at room temperature (RT). The melting process of crystalline ITZ initiated at approximately 169 °C and was completed at around 172 °C. All polymers exhibited an amorphous state under polarized light, and as the temperature increased, the soluplus^®^, HPC, HPMC-AS, and PVP melted between approximately 159 and 210 °C, while only the XL-10 did not melt at all (Figure 6B–F). When the temperature surpassed 170 °C, the ITZ of the PM group exhibited a melting phenomenon. Subsequently, ITZ underwent dispersion into polymers, ultimately resulting in the formation of an ASD. The EXT demonstrated no birefringent phenomenon under polarized light, indicating that the ITZ was dispersed into the polymer matrixes, forming amorphous dispersions. Furthermore, when heated to 170 °C, the melting behavior of the single ITZ was not observed, while the glass transition phenomenon of EXT occurred. The DSC and PLM results indirectly support the amorphous nature of EXT, backing the ASD hypothesis. However, further confirmation through PXRD analysis is necessary to definitively establish the crystal transformation.

#### 3.1.4. PXRD

**Figure 7:**
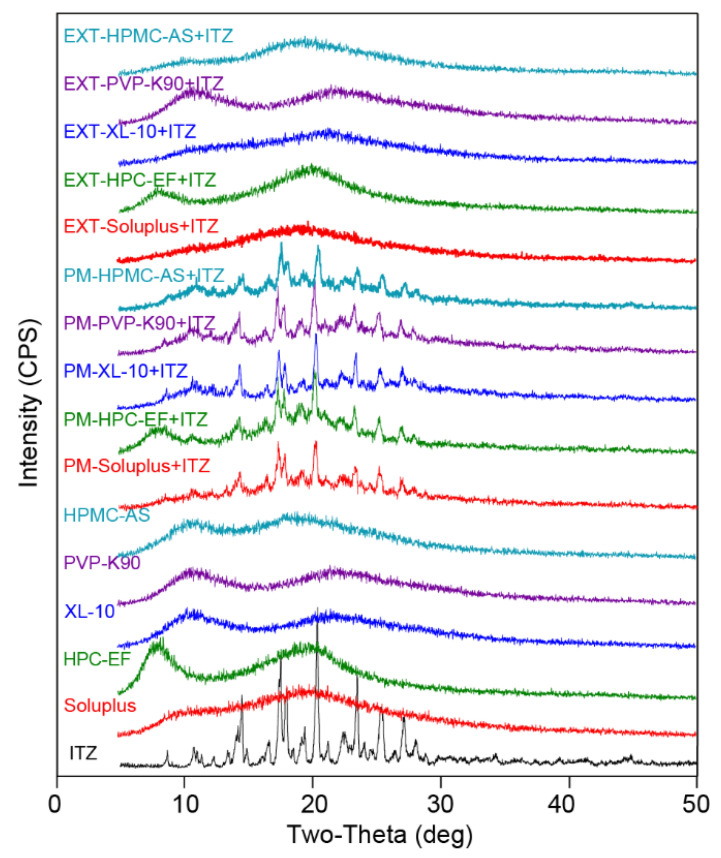
PXRD curves of raw materials and filaments prepared via different methods.

The crystalline state of the ingredients in the formulation was investigated using X-ray powder diffraction, which offers comprehensive insights into the solid-state characteristics of the sample at the atomic level. To validate the conclusion obtained from the DSC curves and PLM figures, PXRD analysis was performed to confirm the crystallinity of ITZ in physical mixtures and extrudates. The extruded filaments were finely ground and sieved through a size 40 mesh before being subjected to XRD analysis. The ITZ exhibited distinct peaks at 11.5, 14.4, 18.2, 20.4, and 23.7 in the 2θ position, as illustrated in Figure 7. These characteristic peaks were also consistently observed in PM. After extrusion, the characteristic peaks of the extrudates disappeared, indicating that ITZ was dispersed into the polymer matrix and formed an amorphous solid dispersion during the HME process. XRD, PLM, and DSC data confirmed the amorphous transformation of the ITZ during HME.

### 3.2. Quality and Quantity of the ITZ

**Figure 8:**
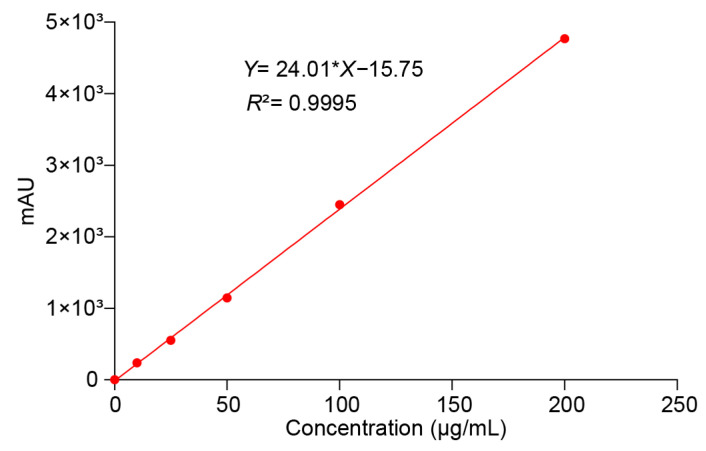
Results of calibration curves, correlation coefficients, and linear ranges of ITZ.

The concentrations of the reference solution were, respectively, 10, 25, 50, 100, and 200 μg/mL. The HPLC conditions specified in Section 2.1.3 were determined. A standard curve was plotted with peak area (y) as the vertical axis and sample amount (x) as the horizontal axis. The results indicated that each component exhibited a good linear relationship within its respective concentration range, as presented in Figure 8.

### 3.3. Formulation and Process Development

#### 3.3.1. Solubility Measurement of ITZ, PM, and EXTs

**Figure 9:**
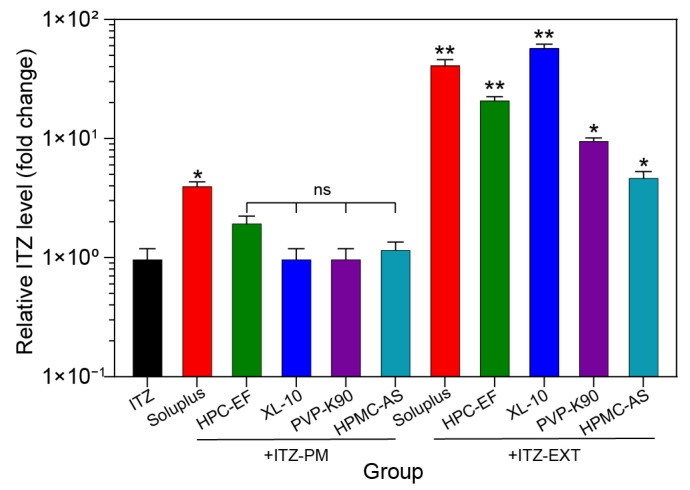
Solubility of ITZ with different polymers prepared by PM and EXT in pH 1.2 medium at RT for 24 h. Significance: ^ns^ *p* > 0.05, * p < 0.05, and ** p <0.01 vs. ITZ.

The solubility of the EXTs’ ITZ was measured at pH 1.2 to assess the impact of various polymer excipients on the solubility of ITZ through ASD. The initial solubility of ITZ was 5.5 μg/mL. In the PM group, most ITZs did not show a significant improvement in solubility, except for soluplus^®,^ which showed a significant enhancement to 22 μg/mL (Table 1). This increase was attributed to that soluplus^®^ can form micelles to solubilize drugs and inhibit the crystallization of drugs in supersaturated solutions, as extensively documented [55,56]. The solubility of ITZ in the EXT group was notably enhanced, reaching values of 236.2 μg/mL (soluplus^®^), 120.0 μg/mL (HPC-EF), 329.1 μg/mL (XL-10), 54.8 μg/mL (soluplus^®^), and 26.8 μg/mL (HPMC-AS), as shown in Figure 9. The experiment revealed that HPMC-AS exhibited pH-dependent solubility, being more soluble at a pH above 5.0, thus limiting the solubility of ITZ in acidic environments [57]. Notably, XL-10, a novel material, increased the solubility of ITZ by 59.3 times. This remarkable increase in solubility suggests that XL-10 has the potential to serve as a new polymer in HME. The reason for this potential lies in its excellent solubilizing ability, which can effectively enhance the bioavailability of drugs by forming ASDs. Moreover, XL-10 has good compatibility and synergy with drugs in the HME process, enabling it to stably disperse drugs and improve their solubility. The results highlight the significant enhancement in ITZ solubility through the preparation of ASD by HME, although further investigation into dissolution rates is warranted through in vitro drug release studies.

#### 3.3.2. In Vitro Drug Release Studies from Extrudates

Given the significant variation in equilibrium solubility of ITZ with different carriers via HME, two dosages (9 mg and 100 mg) were chosen for evaluation in this study. The results showed that the dissolution of ITZ in the PM group was significantly low at both doses, with minimal change observed during the 2 h period, which can be attributed to its low equilibrium solubility. Among the extrudates’ group, all polymers except HPMC-AS demonstrated enhancements in the solubility and dissolution rate of ITZ. The release of ITZ in the EXT group exhibited a gradual increase over extended dissolution periods. Notably, EXT with soluplus^®^ showed an ITZ release exceeding 60% within 5 min. The ITZ in the EXT groups of soluplus^®^ and XL-10 achieved 80% release within 90 min. In contrast, HPMC-AS, being insoluble in acidic environments, did not contribute to the improved solubility of ITZ. Interestingly, this trend was consistent across both the low-dose (equivalent to 9 mg ITZ) and high-dose (equivalent to 100 mg ITZ) groups, suggesting that the drug release rate within each group was predominantly influenced by the characteristics of the respective excipient, rather than being controlled by equilibrium solubility. In order to confirm that the observed phenomenon was not a result of content loss during the HME process, the dissolution test for the high dose (equivalent to 100 mg ITZ) in the EXT groups was extended to 24 h. Samples were collected at 2, 6, 12, 18, and 24 h to monitor the dissolution. Figure S1 illustrates that the EXT groups of XL-10 and soluplus^®^ were fully released within 6 h, while the remaining groups, except for HPMC-AS, dissolved gradually until reaching 100%. The results indicate that ITZ remained stabilized during the HME process and the dissolution rate was influenced by the characteristics of each polymer.

### 3.4. The 3D-Printed Tablets

The preparation of personalized ITZ tablets involves two major steps: first, producing ASD filaments for 3D printing and, then, designing and printing the tablets by means of 3D printing. So, it was necessary to characterize and evaluate the filaments manufactured before subjecting them to the 3D printing process.

The filament composition utilized in the FDM-3D printing comprised 30% ITZ, 65% soluplus^®^, and 5% HPC-EF. The incorporation of 5% HPC-EF was necessary to improve the toughness of the filament, ensuring the smooth execution of the 3D printing process.

#### 3.4.1. Characterization of the 3D Printing Filaments

**Figure 10:**
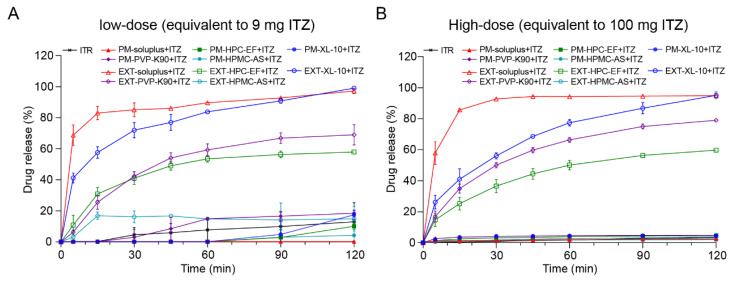
The drug release profiles of ITZ with different polymers prepared by physical mixture (PM) and HME (EXT) in pH 1.2: (**A**) low-dose (equivalent to 9 mg ITZ); (**B**) high-dose (equivalent to 100 mg ITZ).

**Figure 11:**
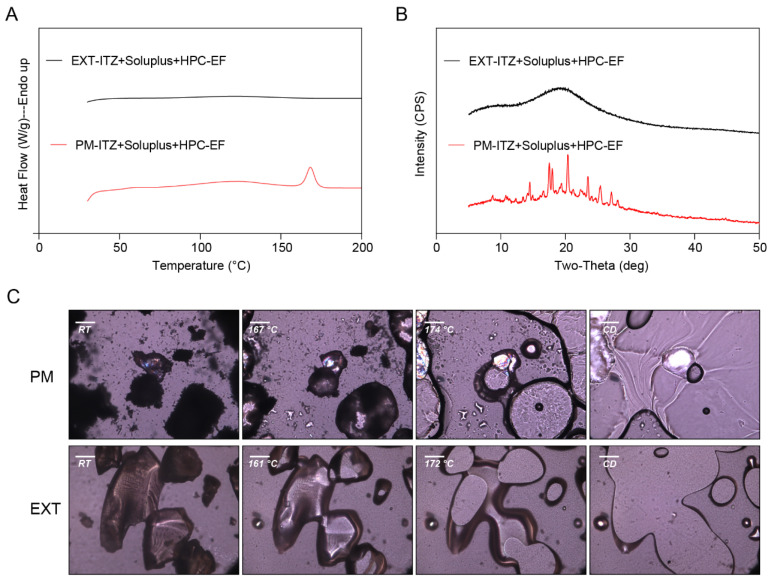
The characterization of ITZ filaments for 3D printing prepared by physical mixture (PM) and HME (EXT): (**A**) DSC curves; (**B**) PXRD curves; and (**C**) PLM pictures. Scale bar: 100 μm.

As illustrated in Figure 10, soluplus^®^ was identified as the primary polymer due to its exceptional ability to enhance solubility. However, according to our previous published works and experiences, filaments composed solely of soluplus^®^ tend to be relatively brittle; thus, in order to obtain ITZ-loaded filaments with adequate physical and mechanical properties for 3D printing, 30% *w*/*w* ITZ was pre-mixed with 65% soluplus^®^ and 5% HPC-EF prepared via the melt extrusion process. Additionally, the solid state of ITZ in extrudates might significantly affect the drug release in the final printed dosages. So, ITZ, DSC, PXRD, and PLM studies were conducted. As shown in Figure 11A, the PM group displayed a diminished melting peak around 168 °C, whereas the EXT group lacked a distinct endothermic peak during the heating process. A similar phenomenon was observed in PLM, where the PM group began to melt at approximately 167 °C. Birefringence could be detected during the melting process until ITZ was completely dissolved in the polymers. In contrast to the PM group, the melting of ITZ and the manifestation of birefringence were not observed in the EXT group, indicating that an ASD was formed (Figure 11C). Furthermore, PXRD was utilized to confirm the formation of an ASD between the ITZ and the polymers (Figure 11B). The characteristic peaks of ITZ are depicted in Figure 7. Notably, these peaks were consistently observed within the PM group, while they disappeared after extrusion, suggesting that ITZ was dispersed within the polymer during the HME process, leading to the formation of an ASD. The combined DSC, PLM, and XRD data provide robust evidence for the amorphous transformation of ITZ during HME.

#### 3.4.2. Evaluation of the 3D-Printed Tablets

The precise dispensing capabilities, spatial control, and layer-by-layer assembly provided by 3D printing enabled the creation of complex compositions and geometries with high accuracy. This technology facilitated the development of dosage forms that incorporated multiple APIs with customized release profiles [58]. Furthermore, the manipulation of tablet dimensions and the number of coating layers was shown to influence the release kinetics of the APIs [53].

In this study, five tablets with distinct inner architectures were designed and produced using 3D printing technology to facilitate a programmed release. Figure 3 illustrates the intricate designs of the tablet structures. The tablets, labeled T1 to T5, were constructed with varying shell thicknesses and infill densities, while maintaining identical overall dimensions of 12 mm in diameter and 4 mm in height. Furthermore, the weights of tablets T1 to T5 were kept consistent, exhibiting a relative weight variation of only 1.45%, which is below the regulatory threshold of 5% [59,60]. The density of the 3D-printed tablets was recorded at less than 1 mg/mm^3^, indicating that these tablets may exhibit buoyancy in solution during the dissolution phase. These characteristics hold potential for the development of floating tablets [61]. Additionally, measurements of the printed tablets confirmed that the dimensional variations between the designed specifications and the actual prints were insignificant (variation < 1.25%), as shown in Table 2. It is noteworthy that most printed dimensions exceeded those of the initial design. The internal structure of the tablets was optimized to ensure that the printed output met a satisfactory quality standard. On one hand, the precision and accuracy for the three dimensions were limited to 0.1 mm, a restriction imposed by the printer technology. Consequently, slight discrepancies may exist between the printed items and their digital counterparts. On the other hand, the printing process has yet to be fully optimized, particularly with respect to cooling rates and platform temperatures. In this investigation, the diameters of the majority of the tablets were larger than intended due to the melting and extrusion processes inherent to 3D printing, which caused the printed tablets to expand during solidification. Similar findings were documented and discussed in our previous research [53,62].

**Figure 12:**
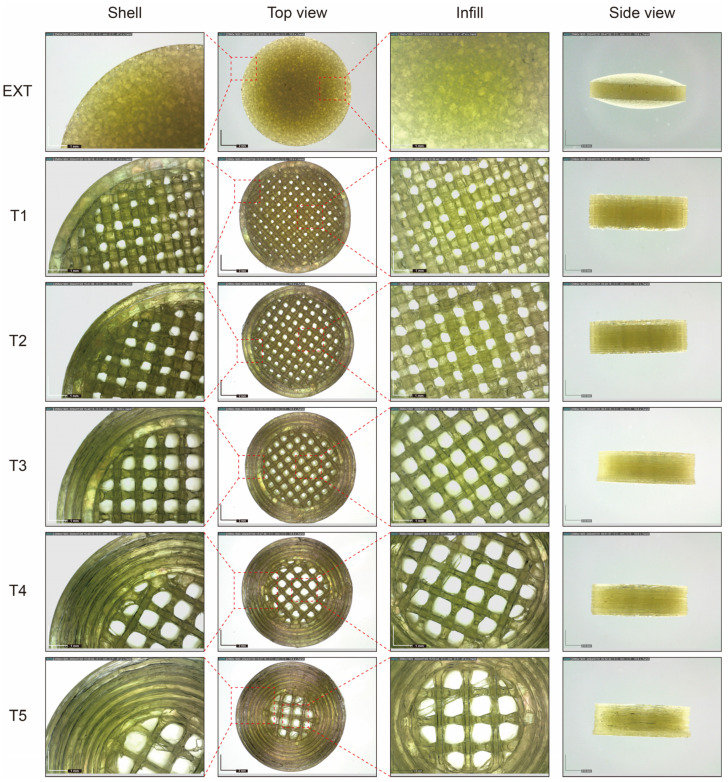
Structure diagram of ITZ tablets. Scale bar: black, 2 mm; white, 1 mm.

To investigate the printed characteristics of the 3D-printed tablets, microscopic imaging techniques were utilized. The properties of the tablets were primarily influenced by the rheological characteristics of the materials employed. As shown in Figure 12, the produced tablets exhibited a notably smoother and more uniform surface. Additionally, this trial indicated elevated hardness values (not displayed), suggesting their potential for delayed dissolution and sustained release effects upon administration. Furthermore, slight variations in the weights and dimensions of the tablets were observed throughout the geometric study, confirming the exceptional reproducibility of the 3D printing process.

#### 3.4.3. In Vitro Drug Release Studies

##### Drug Release

**Figure 13:**
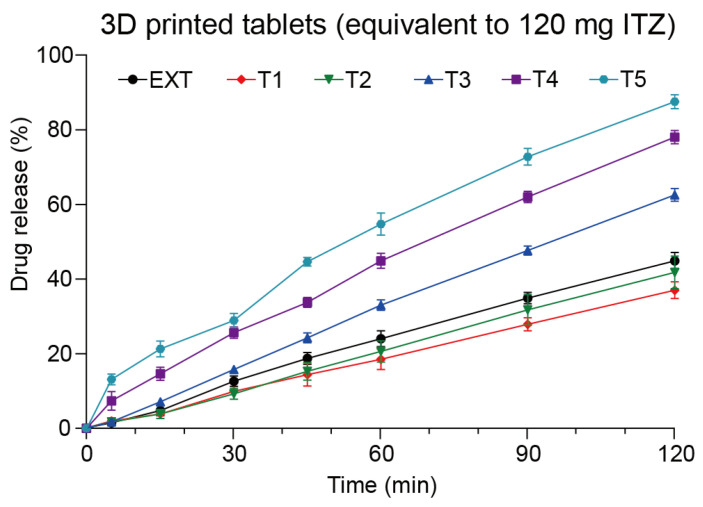
The drug release profiles of direct compressed tablets and 3D-printed tablets.

The in vitro drug release study revealed that the 3D-printed tablets achieved varied release profiles due to differing structural designs. Throughout the dissolution studies, all tablets exhibited no signs of disintegration. Notably, the 3D-printed tablets remained buoyant in the dissolved medium, a crucial factor for enabling controlled release in gastric fluids. This behavior may be attributed to the low density of the 3D-printed tablets (Table 2). As illustrated in Figure 13, the release of ITZ from the 3D-printed tablets appeared to follow zero-order kinetics, indicating that the release rate remained relatively constant throughout the dissolution process. Furthermore, the release rates observed for tablets T1–T5, which shared identical overall dimensions but varied in 3D structure, suggested that drug release can be adjusted by altering the designs. In general, T5 demonstrated the quickest release rate, whereas T1 exhibited the slowest, indicating that a more porous infill structure (less infill) contributes to a higher drug release rate. Additionally, the EXT tablets did not display a significantly faster rate of drug release compared to the 3D-printed tablets, with no substantial increase in ITZ release. As noted in Table 2, the EXT tablets possessed a high density (1.45 mg/mm^3^), which led to their sinking in the medium and ultimately slowed the dissolution rate of ITZ, resulting in only 36% of ITZ released within a 2 h period.

##### The Release Kinetics

During the dissolution studies, it was observed that the printed tablets did not disintegrate at all. The outside layers of the tablets formed a hydrogel interface when in contact with the dissolution medium and then slowly swelled as the medium penetrated into the inner layers of the tablets, thus enabling the ITZ to be slowly released from the hydrogel interface. In addition, as depicted in Figure 13, all the tablets exhibited identical zero-ordered release kinetics. To comprehensively understand the release kinetics from the printed tablets, several mathematical models were employed to analyze the release data, which are outlined in Table 3.

In the present study, the release data for tablets T1–T5 and the direct compressed EXT tablet were evaluated using the previously mentioned models, and the results are summarized in Table 4.

**Figure 14:**
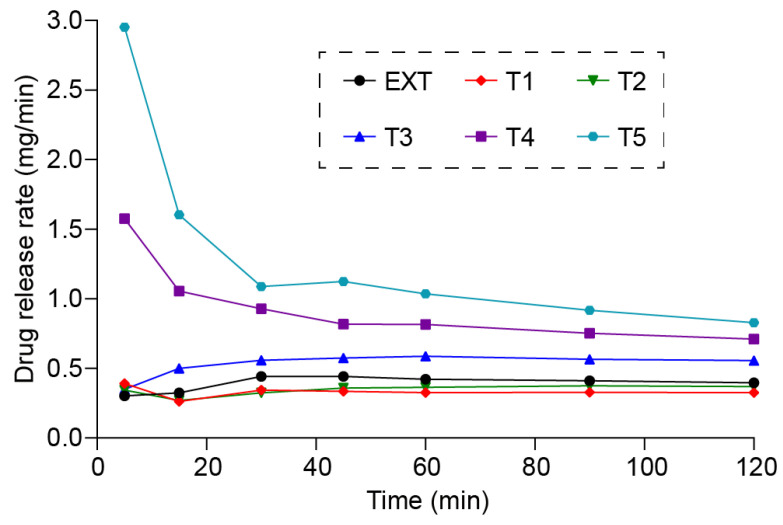
The drug release rates of direct compressed tablets and 3D-printed tablets.

Zero-order, first-order, and Higuchi models were used to determine the release kinetic of the tablets. All the release profiles fit poorly with Higuchi models and were giving R^2^ relatively smaller than the zero- and first-order models, while the zero- and first-order fittings were almost the same, with correlation coefficients exceeding 0.99 and p-values of less than 0.05. Based on the zero-order release model, Figure S2 presents the fitting curve of each tablet. In order to determine the release kinetics, the release drug release rates were calculated utilizing equation 1 and data are plotted in Figure 14, with mg/min verses time.

(1) $$\mathrm{Drug}\;\mathrm{release}\;\mathrm{rate}:\;R_{t}=\frac{M_{t}}{t\;}$$

*R_t_*: The drug release rate at time *t*. *M_t_*: the concentration of drug in the solution at time *t*.

An ideal zero-order release kinetic should have a constant drug release rate, and, as Figure 14 shows, all the tablets had almost a constant drug release rate after 30 min. It took a longer time to reach the steady state for tablets with thicker shells (T5 and T4). Such results indicate that the drug release kinetics followed zero-order kinetics.

Because all the tablets were not disintegrated at all, the Peppas–Sahlin model was applied to further understand whether diffusion or swelling controlled the drug release kinetics. According to the Peppas–Sahlin model, the $k_{1}{*t}^{m}$ represents the Fickian diffusional contribution, whereas the $k_{2}{*t}^{2m}$ represents the Case II relaxational contribution. Thus, the drug release mechanism, characterized by Fickian diffusion and denoted as D, is described by Equation 2. Furthermore, the interplay between the relaxation of the polymeric chains and the influence of Fickian diffusion is illustrated in Equation 3.

(2) $$\mathrm{Fickian}\;\mathrm{diffusion}:\;D=\frac{k_{1}}{k_{1}+k_{2}t^{m}}$$

(3) $$\mathrm{Polymeric}\;\mathrm{chain}\;\mathrm{relaxation}:\;R/F=(k_{2}/k_{1})t^{m}$$

**Figure 15:**
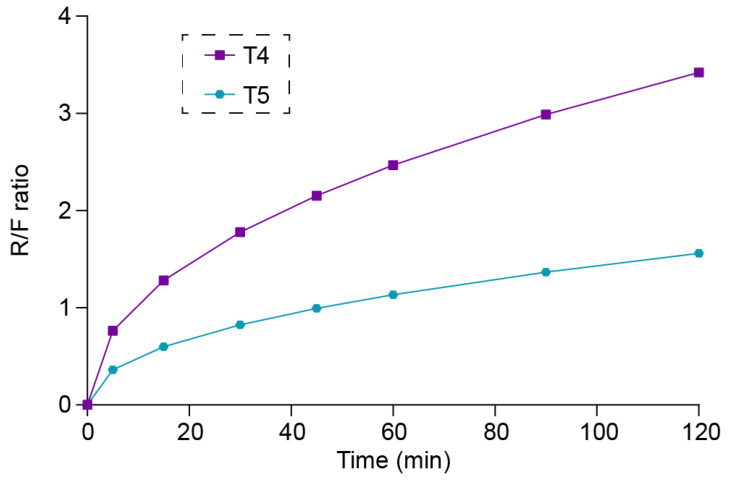
The polymeric relaxation/Fickian diffusion ratio (R/F ratio) curves of T4 and T5 tablets.

As shown in Table 3, the calculated k_1_ values for the T1, T2, T3, and EXT tablets were negative values, which indicates that Fickian diffusion contributed nothing to the release kinetics, while the release kinetics for these tablets were dominated by the polymeric chain relaxation. In addition, the R/F ratio graph (Figure 15) was plotted for tablets T4 and T5. The first 10 min of ITZ released from T4 was dominated by Fickian diffusion, while after 10 min, the release mechanism was dominated by the polymeric chain relaxation. Similarly, the first 45 min of ITZ released from T5 was dominated by Fickian diffusion, while after 45 min, the release mechanism was dominated by the polymeric chain relaxation. And such results also matched the observation from Figure 14, where T4 and T5 took a longer time to reach the steady state.

## 4. Conclusions

This work demonstrated that hot-melt extrusion serves as an effective tool for enhancing the solubility of the poorly water-soluble drug ITZ by forming ASDs with a series of hydrophilic or amphiphilic carbohydrate polymers. Five different polymers were employed for formulation screening. The ASD formulation with ITZ in soluplus^®^ and Plasidon XL-10 polymers exhibited the most significant solubility improvement, being 42.6 and 59.3 times that of the raw ITZ, respectively. Moreover, the ITZ-loaded filament was prepared via the HME process and was suitable for the general FDM-3D printing process. Additionally, tablets with different structures were designed and successfully printed via 3D printing. Solid-state analysis and morphology studies were conducted prior to in vitro studies. The results indicated that ASD tablets were successfully manufactured and the tablet quality was adequate. The in vitro drug release studies showed that the drug release rate and mechanisms can be manipulated by simply altering the structural design. Additionally, the release studies also provided a comprehensive understanding of the impact of geometry design on the drug release kinetics and drug release profiles. The aforementioned findings broaden the application of ITZ in curing systemic infectious diseases and offer a new solution to improve solubility using crosslinked polymers such as soluplus^®^ and Plasidon XL-10. In addition, this work also proved that combining HME and FDM-3D printing for the on-demand manufacturing of patient-focused drug products could be an optimal approach for future pharmaceutical development.

## Figures and Tables

**Table 1:** Variation in aqueous solubility in pH 1.2 at room temperature for 24 h. Significance: * p < 0.05 and ** p < 0.01 vs. ITZ.

Group	Solubility (μg/mL)	Times
ITZ	5.5	1.0
PM-soluplus^®^ + ITZ	22.9	4.1 *
PM-HPC-EF + ITZ	8.4	1.5
PM-XL-10 + ITZ	11.1	2.0
PM-PVP-K90 + ITZ	5.6	1.0
PM-HPMC-AS + ITZ	6.7	1.2
EXT-soluplus^®^ + ITZ	236.2	42.6 **
EXT-HPC-EF + ITZ	119.9	21.6 **
EXT-XL-10 + ITZ	329.1	59.3 **
EXT-PVP-K90 + ITZ	54.8	9.9
EXT-HPMC-AS + ITZ	26.8	4.8

**Table 2:** Geometric characteristics of the 3D-printed tablets.

#	Shell (mm)	Infill %	Diameter (mm)	* Variation %	Height (mm)	* Variation %	Weight (mg)	** Variation %	Density (mg/mm^3^)
EXT	0	100	12.00 ± 0.01	0.00	2.45 ± 0.01	0.24	401.67 ± 3.79	0.94	1.45
T1	0.4	90	12.08 ± 0.03	0.69	3.98 ± 0.04	1.25	397.63 ± 5.33	1.34	0.87
T2	0.8	82	12.07 ± 0.01	0.56	3.98 ± 0.06	0.75	392.83 ± 2.42	0.62	0.87
T3	1.6	76	12.09 ± 0.04	0.78	3.99 ± 0.08	1.75	401.23 ± 2.17	0.54	0.87
T4	2.4	68	12.06 ± 0.03	0.50	3.97 ± 0.03	0.25	402.37 ± 5.90	1.47	0.88
T5	3.2	60	12.09 ± 0.02	0.72	3.99 ± 0.01	0.25	412.07 ± 5.98	1.45	0.90

* The variations in diameter and height were calculated using the following equation: $Variation\%=\frac{D_{m}-D_{d}}{D_{d}}*100\%$ where $D_{m}$ is the measured diameter or height of the printed tablets, while $D_{d}$ is the designed diameter or height of the digital tablet models. ** The variation in weight was calculated using the following equation: $Variation\;\%=\frac{S.D.}{Average}*100\%\;$where S.D. is the standard error of all six measured tablets, while Average is the average value of all six measured tablets.

**Table 3:** The formula and definition of different mathematical models.

Mathematical Models	Formula	Definition
Zero-order	$Q_{t}=Q_{0}+K_{0}*t$	*Q_t_*: the amount of drug released in time *t* *Q*_0_: the initial amount of drug in the solution *K*_0_: the zero-order release constant
First-order	$\log C=\log C_{0}-kt/2.303$	*C*: the amount of drug at time *t* $C_{0}$: the initial concentration *k*: the first-order rate constant
Higuchi	$\frac{Q_{t}}{Q_{\infty }}=k\;{*\;t}^{1/2}$	*Q_t_*: the drug released at time *t* $Q_{\infty }$: the drug loading of the dosage *K*: Higuchi constant
Korsmeyer–Peppas	$\frac{Q_{t}}{Q_{\infty }}=k\;{*\;t}^{n}$	*Q_t_*: the drug released at time *t* $Q_{\infty }$: the drug loading of the dosage *k*: the rate constant *n*: the release exponent
Peppas–Sahlin	$\frac{Q_{t}}{Q_{\infty }}=k_{1}\;{*\;t}^{m}+k_{2}\;{*\;t}^{2m}$	*Q_t_*: drug released at time *t* $Q_{\infty }:$ is the drug loading of the dosage *k*_1_/*k*_2_: the kinetic constant *m*: the release exponent

**Table 4:** The kinetic constant and correlation coefficients of the different models.

#	Zero-Order		First-Order		Higuchi		Korsmeyer–Peppas			Peppas–Sahlin			
	K_0_	R^2^	k	R^2^	k	R^2^	k_kp_	n	R^2^	k_1_	k_2_	m	R^2^
EXT	0.003	0.9963	0.000031	0.9963	0.027	0.8722	0.004	0.949	0.9973	−0.021	0.014	0.373	0.9991
T1	0.002	0.9989	0.000025	0.9989	0.021	0.8601	0.003	0.994	0.9989	−0.002	0.003	0.473	0.9990
T2	0.003	0.9975	0.000031	0.9974	0.024	0.8361	0.002	1.061	0.9988	−0.005	0.004	0.476	0.9991
T3	0.004	0.9988	0.000042	0.9988	0.037	0.8603	0.004	0.989	0.9988	−0.019	0.012	0.410	0.9998
T4	0.006	0.9814	0.000055	0.9818	0.049	0.9248	0.016	0.812	0.9992	0.014	0.005	0.472	0.9995
T5	0.006	0.9411	0.000065	0.9417	0.058	0.9563	0.024	0.703	0.9946	0.029	0.005	0.460	0.9955

## Data Availability

The original contributions presented in the study are included in the article/Supplementary Material, further inquiries can be directed to the corresponding author.

## Supplementary Materials

The following supporting information can be downloaded at: https://www.mdpi.com/article/10.3390/polym16233302/s1, Figure S1. The drug release profiles of ITZ (high-dose) with different polymers prepared by HME (EXT) in pH 1.2 for 24 h. Figure S2. Results of fitting the dissolution of 3D printed tablets according to the zero-order model.
