# Supplementary material for: Improve Solubility and Develop Personalized Itraconazole Dosages via Forming Amorphous Solid Dispersions with Hydrophilic Polymers Utilizing HME and 3D Printing Technologies
Source: Polymers (Basel). 2024 Nov 26;16(23):3302. doi: 10.3390/polym16233302 (PMC11644310; doi:10.3390/polym16233302)
Supplement: Supplementary file 1 [file polymers-16-03302-s001.zip › polymers-3292821-supplementary.pdf]

**Supplementary data**

Figure S1. The drug release profiles of ITZ (high-dose) with different polymers prepared by HME (EXT) in pH 1.2 for 24 h.

Figure S2. Results of fitting the dissolution of 3D printed tablets according to the zero-order model.

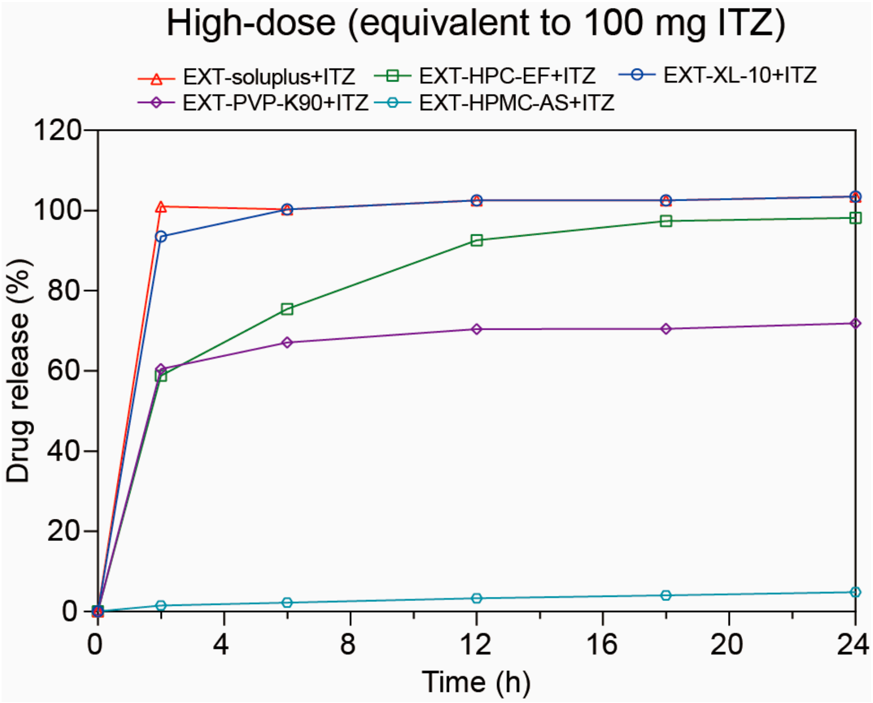


**Figure S1**. The drug release profiles of ITZ (high-dose) with different polymers prepared by HME (EXT) in pH 1.2 for 24 h.

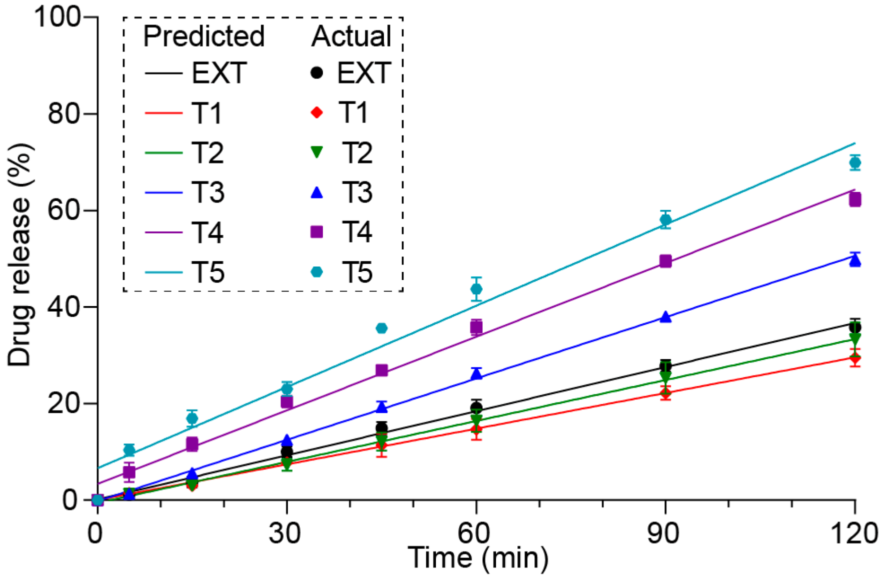


**Figure S2.** Results of fitting the dissolution of 3D printed tablets according to the zero-order model.
